# Genome-Wide DNA Methylation Profile in Jejunum Reveals the Potential Genes Associated With Paratuberculosis in Dairy Cattle

**DOI:** 10.3389/fgene.2021.735147

**Published:** 2021-10-15

**Authors:** Junnan Zhang, Bo Han, Weijie Zheng, Shan Lin, Houcheng Li, Yahui Gao, Dongxiao Sun

**Affiliations:** Key Laboratory of Animal Genetics, Breeding and Reproduction of Ministry of Agriculture and Rural Affairs, National Engineering Laboratory for Animal Breeding, Department of Animal Genetics, Breeding and Reproduction, College of Animal Science and Technology, China Agricultural University, Beijing, China

**Keywords:** paratuberculosis, DNA methylation, candidate gene, regulatory mechanism, whole-genome bisulfite sequencing, dairy cattle

## Abstract

Paratuberculosis in cattle causes substantial economic losses to the dairy industry. Exploring functional genes and corresponding regulatory pathways related to resistance or susceptibility to paratuberculosis is essential to the breeding of disease resistance in cattle. Co-analysis of genome-wide DNA methylation and transcriptome profiles is a critically important approach to understand potential regulatory mechanism underlying the development of diseases. In this study, we characterized the profiles of DNA methylation of jejunum from nine Holstein cows in clinical, subclinical, and healthy groups using whole-genome bisulfite sequencing (WGBS). The average methylation level in functional regions was 29.95% in the promoter, 29.65% in the 5’ untranslated region (UTR), 68.24% in exons, 71.55% in introns, and 72.81% in the 3’ UTR. A total of 3,911, 4,336, and 4,094 differentially methylated genes (DMGs) were detected in clinical vs. subclinical, clinical vs. healthy, and subclinical vs. healthy comparative group, respectively. Gene ontology (GO) and analysis based on the Kyoto Encyclopedia of Genes and Genomes (KEGG) showed that these DMGs were significantly enriched in specific biological processes related to immune response, such as Th1 and Th2 cell differentiation, wnt, TNF, MAPK, ECM-receptor interaction, cellular senescence, calcium, and chemokine signaling pathways (*q* value <0.05). The integration of information about DMGs, differentially expressed genes (DEGs), and biological functions suggested nine genes *CALCRL*, *TNC*, *GATA4*, *CD44*, *TGM3*, *CXCL9*, *CXCL10*, *PPARG,* and *NFATC1* as promising candidates related to resistance/susceptibility to Mycobacterium avium subspecies paratuberculosis (MAP). This study reports on the high-resolution DNA methylation landscapes of the jejunum methylome across three conditions (clinical, subclinical, and healthy) in dairy cows. Our investigations integrated different sources of information about DMGs, DEGs, and pathways, enabling us to find nine functional genes that might have potential application in resisting paratuberculosis in dairy cattle.

## Introduction

Animal health, as a main factor affecting the development of the animal husbandry economy, is being valued progressively more over time and incorporated into breeding programs. Paratuberculosis is referred to as Johne’s disease and has been reported all over the world bringing huge economic losses to the dairy industry, warranting more attention (Ott et al., 1999). The disease is a chronic debilitating enteritis caused by a *Mycobacterium avium* subspecies *paratuberculosis* (MAP) infection (Clarke, 1997), which has a long incubation period. Clinical manifestations contain chronic or intermittent diarrhea, throat and jaw edema, weight loss, and eventually death (Whitlock and Buergelt, 1996). Verschoor et al., 2010 reported that four single nucleotide polymorphisms (SNPs) in the *IL10RA* gene were significantly associated with paratuberculosis susceptibility in the Canadian Holstein cattle population (Verschoor et al., 2010). Hempel et al., 2016 identified seven genes, *FABP6*, *SLC10A2*, *MMP13*, *APOB*, *IGSF23*, *GNLY,* and *FCRLA*, related to paratuberculosis through RNA sequencing (RNA-Seq) of ileocecal tissues from Holstein cows (Hempel et al., 2016). Studies have proved that jejunum is the main invasion and residence site of MAP, the infected jejunum wall is diffusely thickened, showing inflammation and tissue edema. The enlargement of mesenteric and other regional lymph nodes are usually apparent (Wentink et al., 1994; Bannantine and Bermudez, 2013; Ibeagha-Awemu et al., 2019). Therefore, we also used jejunum tissue to explore the mechanism of paratuberculosis diseases in our study.

The genome-wide DNA methylation genetic map is an effective strategy to reveal the role of DNA in development and disease. It has been reported that abnormal DNA methylation is related to human diseases, such as cancer and neurodevelopmental diseases (Weksberg et al., 2001; Debaun et al., 2003). Prawitt et al. (2005) found that aberrant methylation at the *ICR1* gene led to human Wilms’ tumor and Beckwith–Wiedemann syndrome. Furthermore, a growing number of studies are considering the use of epigenomics to study disease resistance in livestock (Moore et al., 2008; Jin et al., 2011; Hao et al., 2016; Sharifi-Zarchi et al., 2017; Fu et al., 2018). Whole-genome bisulfite sequencing (WGBS) is used to determine the DNA methylation status of single cytosines by treating the DNA with bisulfite before sequencing (Stevens et al., 2013). Compared with the previous methylation sequencing technology, WGBS requires a smaller sample volume and gets a resolution off a single base, which may detect the methylation status of each cytosine.

In our previous studies, we detected the serum antibody levels of MAP based on an enzyme-linked immunosorbent assay (ELISA) for 8,214 Chinese Holstein cows and estimated the heritability of susceptibility to paratuberculosis (0.04–0.11) (Gao et al., 2018a). We further identified 26 genome-wide significant SNPs and 10 functional genes associated with paratuberculosis by performing a genome-wide association study, namely *IL-4*, *IL-5*, *IL-13*, *IRF1*, *MyD88*, *PACSIN1*, *DEF6*, *TDP2*, *ZAP70,* and *CSF2* (Gao et al., 2018b). In addition, we conducted RNA sequencing on the jejunum samples from MAP-infected and healthy Holstein cows and identified 134 differentially expressed genes among clinical, subclinical, and healthy groups (unpublished data). However, research related to the regulatory roles of DNA methylation on paratuberculosis in dairy cattle has not been reported until now. In the present study, WGBS was using with the same samples as those for our RNA-Seq study to investigate the regulatory mechanism of DNA methylation on paratuberculosis and identify potentially critical genes for resistance/susceptibility to MAP in order to provide molecular information for a disease-resistance breeding program in dairy cattle.

## Methods and Materials

### Sample Collection

In our previous studies (Gao et al., 2018a; Gao et al., 2018b), we detected the serum antibody levels for MAP of 8,214 Chinese Holstein cows with the ELISA method, of which 784 cows were identified as positive. Stool samples of each seropositive cow were further collected for quantitative PCR (qPCR) to detect whether MAP was present with an INgene q ParaTB Kit (Ingenasa, Madrid, Spain). Individuals who were positive in both the serum and stool samples and had obvious diarrhea were defined as clinical cows (CC); individuals who were seropositive, but their stools were negative and with no diarrhea, were referred to as subclinical cows (SC); individuals that were negative in both serum and stools samples were treated as healthy cows (HC). Three cows were included in each group (Supplementary Figure S1A). These nine cows were dissected in the same slaughterhouse. The jejunum tissues were collected from each individual within 30 min after slaughtering and placed in liquid nitrogen. The infected part of the jejunum from clinical cows was obviously thickened, inflamed, and edema was present, and that from subclinical cows showed mild inflammation and edema.

### Deoxyribonucleic Acid Extraction and Library Preparation

Genomic DNA of the jejunum tissue from the nine cows with three in each group was extracted using a TIANamp Genomic DNA Kit (Tiangen, Beijing, China). The DNA quantity and quality were determined using a NanoDrop2000 spectrophotometer (ThermoFisher, Waltham, MA, United States) and Agilent 2100 Bioanalyzer (Agilent, Santa Clara, CA, United States), respectively.

First, 5 μg of lambda DNA was added into the genomic DNA of each sample as the negative control. Then, the genomic DNA was randomly broken into 200–300 bp fragments using an S220 focused-ultrasonicator (Covaris, Santa Clara, MA, United States); these fragments were end-repaired, added to the sequencing adapter, and treated with bisulfite. The methylated cytosines were thereby unchanged and the unmethylated cytosines became uracils, which were changed to thymines after PCR amplification (Supplementary Figure S1B). A library quality control was performed with an Agilent 2100 Bioanalyzer.

### Bisulfite Sequencing and Data Analysis

With the completion of the library, paired-end sequencing was performed on the Illumina NovaSeq6000 sequencing platform (Illumina, CA, United States). Preliminary quality control of raw reads was carried out with fastqc (v0.11.9, https://www.bioinformatics.babraham.ac.uk/projects/fastqc/) and these reads were then filtered with fastp software (v0.20) to remove adapters and low quality sequences (Chen et al., 2018). With bowtie2 (v2.4.0), clean reads were deduplicated and aligned against the bovine reference genome (ARS-UCD1.2, https://asia.ensembl.org/index.html), which was bisulfite-converted using bismark (v0.22.1) (Langmead and Salzberg, 2012; Langmead et al., 2018), and the command of “bismark--genome_folder ref.fa -bowtie2 -N 0 -L 20”. Methylation status was determined and methylated CpG sites were marked using the command “bismark_methylation_extractor” (Krueger and Andrews, 2011). The correlation coefficient within each group was calculated using R programing language (v3.6.0).

### Identification of Differentially Methylated Regions and Differentially Methylated Genes

The bsseq (v1.24.4) package in the R programing language (v3.6.0) was used to find differentially methylated regions (DMRs) among the three comparative groups based on the information about CpG sites (Hansen et al., 2012). Each DMR had at least five methylated CpG sites where the distance between CpG sites was less than 300 bp and there was a greater than 10% difference in methylation levels between groups. The significant ranking of DMRs was dependent on the absolute value of areaStat that was used to determine the significance threshold. In order to screen differentially methylated genes (DMGs), we retained the genes in which the DMRs were entirely located using the “intersect–f 1” command from bedtools (v2.28.0) (Quinlan and Hall, 2010), eliminating genes that had partial DMRs, all these screened genes were defined as DMGs. The promoter is located kilo bases upstream away from the transcriptional start site in the regulatory sequence controlling gene expression (Hernandez-Garcia and Finer, 2014; Yu et al., 2016). Therefore, we defined 2000 bp upstream of the gene as the promoter region and identified the differentially promoter-methylated genes (DPMGs) with identical screening. Both the files of the gene body and promoter were downloaded from UCSC (https://genome.ucsc.edu/). The genes containing both hypermethylated and hypomethylated regions were considered as hypermethylated and hypomethylated genes.

### Gene Ontology and Kyoto Encyclopedia of Genes and Genomes Functional Enrichment Analysis

The clusterProfiler tool (v3.16.0) in R was applied to perform GO and KEGG enrichment analysis on DMGs and DPMGs (Yu et al., 2012). The significant threshold related to enrichment analysis of DMGs and DPMGs was a *q* value <0.05 and *p* value <0.05, respectively. The *p* value is a measure of statistical significance; the *q* value is a measure of the false discovery rate (FDR) (Benjamini and Hochberg, 1995; Storey, 2003).

### Integration Analysis of Whole-Genome Bisulfite Sequencing and Ribonucleic Acid Seq Data

Genes that were both DMGs and DEGs according to the DNA methylation and RNA sequencing data were identified. Further, we detected the methylated status of promoters in these genes and calculated the association between the methylated status and the fragments per kilobase million (FPKM) with Pearson correlation analysis (Schober et al., 2018).

## Results

### Summary of Methylome Sequencing

By performing WGBS, the raw data of the nine jejunum tissue samples from clinical, subclinical, and healthy groups, each comprising three Holstein cows were completed. After quality control, a total of 2.84 × 10^9^ clean reads were obtained with an average of 3.16 × 10^8^ (average 108.33 G per sample) for each sample, indicating a sequencing depth of 30×. There were 81.03% of non-duplicated clean reads uniquely aligned to the bovine reference genome (ARS-UCD1.2, Supplementary Table S1). Bisulfite conversion efficiency (BCE) reached 99.33% suggesting the reliability of the methylome sequencing in this study (Table 1).

**TABLE 1:** Basic state of alignment, bisulfite conversion efficiency, and methylation level.

Group	BCE (%)	Unique alignment (%)	Methylation level (%)	Methylation level (by group)
CC1	99.33%	81.60%	67.41%	67.42%
CC2	99.33%	80.40%	66.78%	
CC3	99.33%	80.90%	68.06%	
SC1	99.32%	81.30%	65.29%	66.74%
SC2	99.36%	81.40%	67.50%	
SC3	99.34%	80.00%	67.42%	
HC1	99.33%	79.70%	63.36%	65.26%
HC2	99.34%	81.00%	65.00%	
HC3	99.33%	80.00%	67.44%	

Note: CC: clinical cow; SC: subclinical cow; HC: healthy cow; and BCE: bisulfite conversion efficiency.

### Deoxyribonucleic Acid Methylation Patterns

We found that on average 3.22% of all genomic C sites were methylated across the nine samples. Methylation in advanced mammals generally exists in three sequence contexts: CpG, CHG (where H is A, C, or T), and CHH. Here, we observed the overall genome-wide methylation levels of 70.50% CpG, 0.40% CHG, and 0.38% CHH in the clinical group, 69.60% CpG, 0.39% CHG, and 0.38% CHH methylation in the subclinical group, and 68.03% CpG, 0.39% CHG, and 0.38% CHH methylation in the healthy group (Supplementary Table S1). Further, we calculated the correlation of 0.9 within each group using the methylation level of CpGs.

### Deoxyribonucleic Acid Methylation Levels in Different Regions of the Gene

**FIGURE 1:**
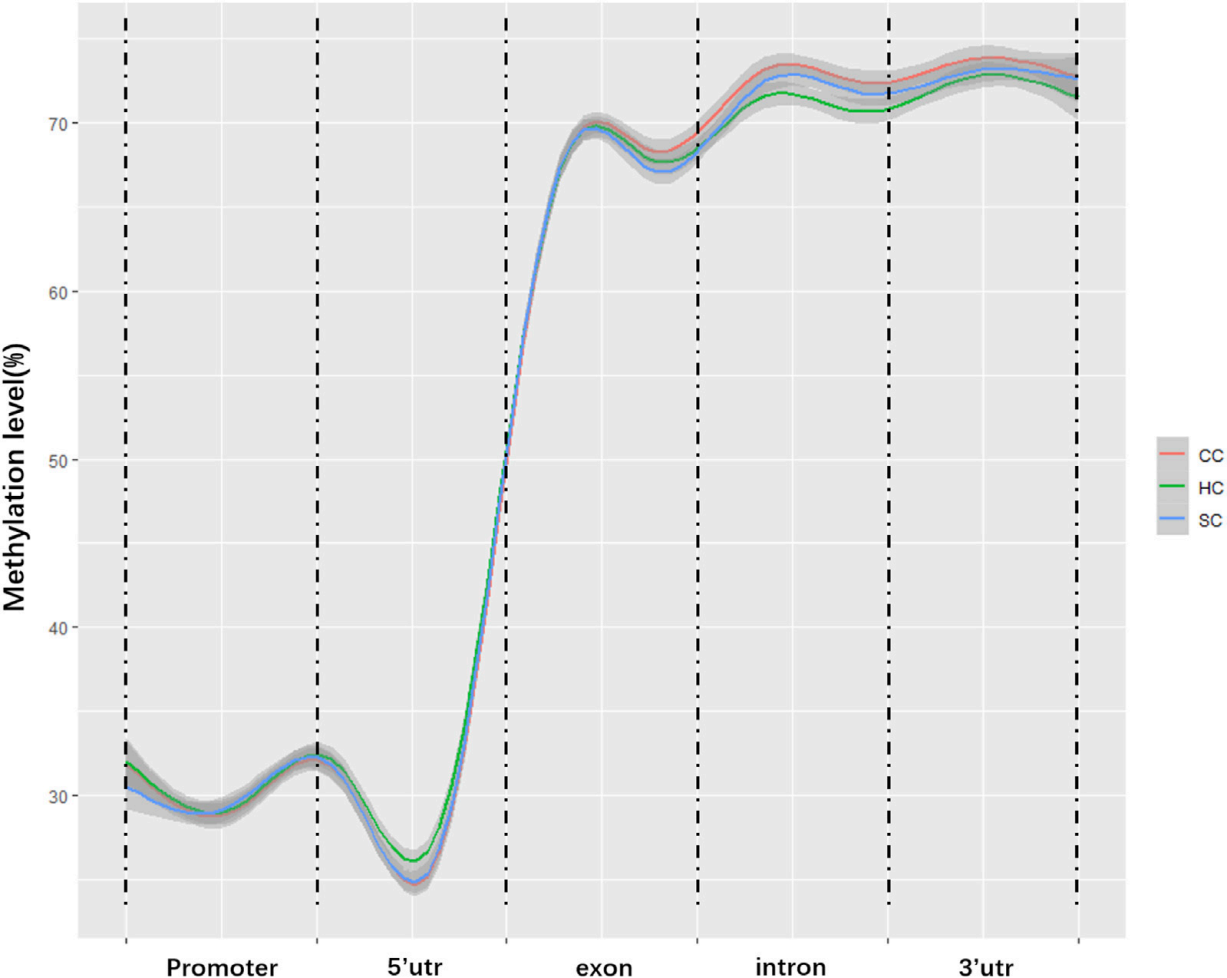
Average methylation levels in different genomic regions. The *y*-axis is the methylation level; the *x*-axis is the different regions in the genome. CC: clinical cow; SC: subclinical cow; and HC: healthy cow.

**FIGURE 2:**
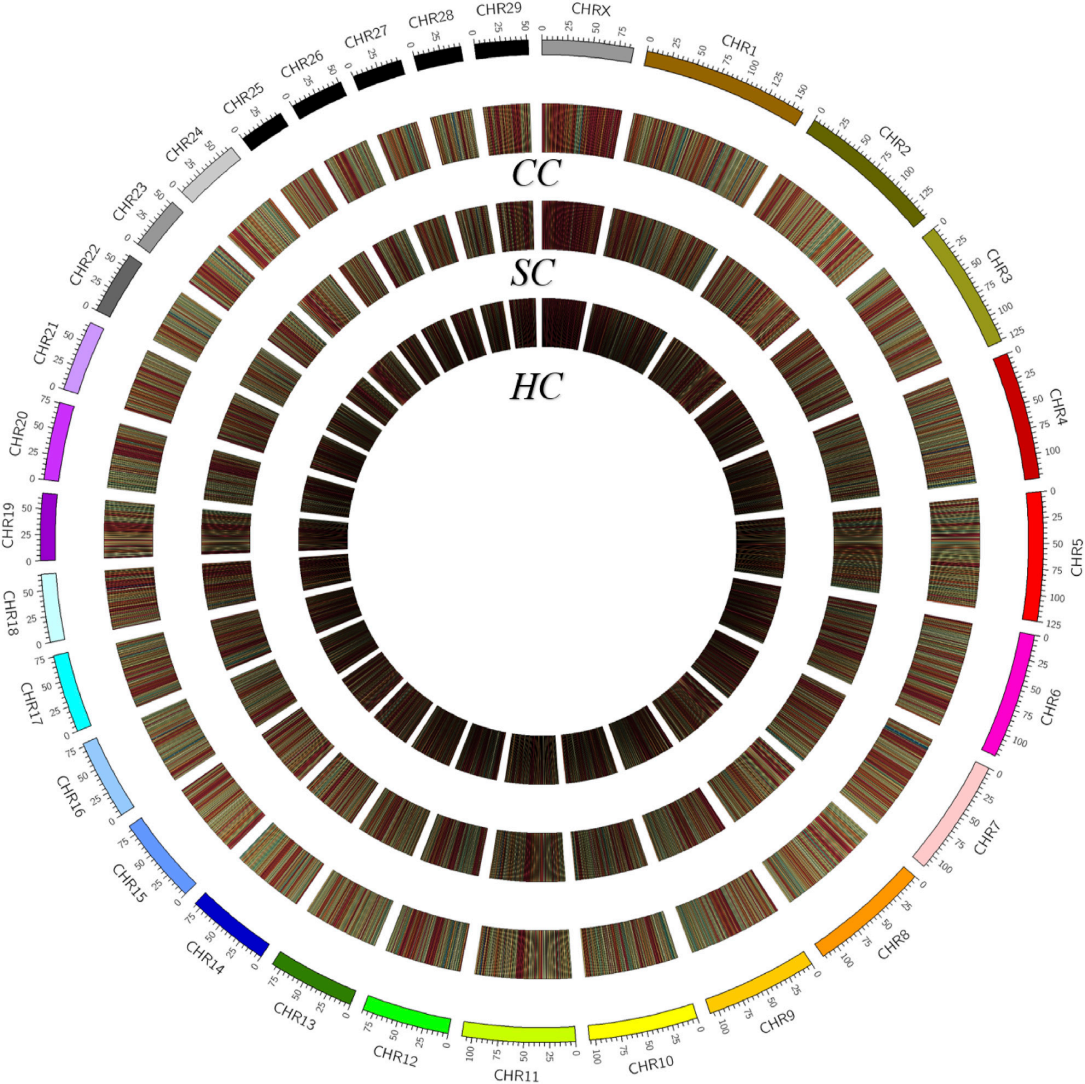
Density plot of 5-methylcytosine among various groups. Chromosome numbers and scales are indicated on the periphery, a dark to light color indicates a low to high level of methylation. CC: clinical cow; SC: subclinical cow; and HC: healthy cow.

To characterize the distribution of methylation in different genomic elements, we analyzed the average DNA methylation levels in the promoter, 5’ untranslated region (UTR), exons, introns, and the 3’ UTR of each methylated gene, and found the average methylation levels of the three experimental groups were similar in the promoter (29.3–30.48%) and 5’ UTR (29.02–30.29%) but displayed certain differences in partial exons, introns, and 3’UTR (Figure 1). Of note, the methylation levels of partial exons, introns, and the 3’UTR in the clinical group (68.50, 72.40, and 73.35%) were obviously higher than those in the healthy groups (67.90, 70.60, and 71.22%). In addition, the methylation level in the X chromosome (63.45%) was significantly lower than that in the autosomes (69.65%, *p* value <0.05; Figure 2).

### Differentially Methylated Regions, Differentially Methylated Genes, and Differentially Promoter-Methylated Genes Identification

By comparing the cytosine methylation profiles of MAP-infected and healthy groups, we detected 100,758 differentially methylated regions (DMRs), including 31,750 between the clinical and subclinical groups, 35,082 between the clinical and healthy groups, and 33,926 between the subclinical and healthy groups (Supplementary Table S2). The length of DMRs was 6–2,292 bp with an average of 350 bp, 98.5% of which were less than 1,000 bp (Supplementary Figure S2). Further, a total of 6,653 differentially methylated genes (DMGs) and 1,779 differentially promoter-methylated genes (DPMGs) were identified, i.e., 3,911 DMGs and 661 DPMGs in clinical vs. subclinical groups, 4,336 DMGs and 893 DPMGs in clinical vs. healthy groups, and 4,094 DMGs and 746 DPMGs in subclinical vs. healthy groups (Supplementary Table S2). Details of the top 10 significant DMGs and DPMGs in the three comparisons are described in Table 2 and Table 3, respectively.

**TABLE 2:** The top 10 DMGs among clinical, subclinical, and healthy groups.

Group	Gene	Full name	areaStat
CC vs.SC	*ABCA9*	ATP binding cassette subfamily A member 9	−207.03
	*IGF1R*	insulin like growth factor 1 receptor	−202.65
	*IRF2*	interferon regulatory factor 2	178.43
	*TRIM36*	tripartite motif containing 36	170.80
	*ATP2B2*	ATPase plasma membrane Ca2+ transporting 2	161.54
	*ZNF521*	zinc finger protein 521	−155.53
	*COMT*	catechol-O-methyltransferase	−146.49
	*MAP2K6*	mitogen-activated protein kinase kinase 6	−139.39
	*GLUD1*	glutamate dehydrogenase 1	−136.60
	*KCNU1*	potassium calcium-activated channel subfamily U member 1	−133.99
CC vs.HC	*TRIM9*	tripartite motif containing 9	−206.52
	*VGLL4*	vestigial like family member 4	195.48
	*ZNF521*	zinc finger protein 521	−189.58
	*FGF12*	fibroblast growth factor 12	−171.36
	*AP1M1*	adaptor related protein complex 1 subunit mu 1	−166.31
	*MSX2*	msh homeobox 2	−158.98
	*SPART*	spartin	−147.53
	*LITAF*	lipopolysaccharide induced TNF factor	−136.44
	*PRKN*	parkin RBR E3 ubiquitin protein ligase	135.78
	*UBE2V1*	ubiquitin conjugating enzyme E2 V1	132.49
SC vs.HC	*FNTB*	farnesyltransferase, CAAX box, beta	181.33
	*GLUD1*	glutamate dehydrogenase 1	147.12
	*FARS2*	phenylalanyl-tRNA synthetase 2, mitochondrial	−142.08
	*GTF2IRD1*	GTF2I repeat domain containing 1	−141.27
	*IQCA1*	IQ motif containing with AAA domain 1	−130.13
	*CCDC88B*	coiled-coil domain containing 88B	−129.05
	*RPH3AL*	rabphilin 3A like (without C2 domains)	−128.24
	*UBE2V1*	ubiquitin conjugating enzyme E2 V1	125.99
	*OAS1Y*	2’,5’-oligoadenylate synthetase 1, 40/46 kDa	−123.96

Note: DMGs: differentially methylated genes; CC: clinical cow; SC: subclinical cow; HC: healthy cow; vs.: versus; areaStat: the sum of the t-statistics in each CpG, the magnitude of its absolute value represents the degree of methylation difference, the positive value represents hypermethylation, and the negative value represents hypomethylation.

**TABLE 3:** The top 10 DPMGs among clinical, subclinical, and healthy groups.

Group	Gene	Full name	areaStat
CC vs.SC	*KBTBD6*	kelch repeat and BTB domain containing 6	134.82
	*DOLK*	dolichol kinase	97.46
	*SPATA7*	spermatogenesis associated 7	−81.91
	*DCTN1*	dynactin subunit 1	−80.92
	*CDKN1B*	cyclin dependent kinase inhibitor 1B	−80.63
	*STAG2*	stromal antigen 2	74.02
	*ALKBH1*	alkB homolog 1, histone H2A dioxygenase	69.09
	*SMN2*	survival of motor neuron 2, centromeric	66.22
	*TRAPPC12*	trafficking protein particle complex 12	64.98
	*BAG3*	BAG cochaperone 3	−62.08
CC vs.HC	*NEUROD1*	neuronal differentiation 1	−141.99
	*DOLK*	dolichol kinase	126.79
	*CDH10*	cadherin 10	−108.22
	*TMEM47*	transmembrane protein 47	−107.76
	*USP44*	ubiquitin specific peptidase 44	−104.73
	*KCNH5*	potassium voltage-gated channel subfamily H member 5	−100.64
	*SPATA7*	spermatogenesis associated 7	−97.40
	*NFYB*	nuclear transcription factor Y subunit beta	−95.71
	*TMEM184A*	transmembrane protein 184A	−94.14
	*BLOC1S4*	biogenesis of lysosomal organelles complex 1 subunit 4	−91.18
SC vs.HC	*THOC2*	THO complex 2	−144.59
	*NEUROD1*	neuronal differentiation 1	−116.99
	*DNAJA1*	DnaJ heat shock protein family (Hsp40) member A1	−102.50
	*SOX5*	SRY-box transcription factor 5	−96.20
	*NRK*	Nik related kinase	−92.11
	*MRPS24*	mitochondrial ribosomal protein S24	−87.19
	*USP44*	ubiquitin specific peptidase 44	−82.56
	*CHCHD8*	coiled-coil-helix-coiled-coil-helix domain containing 8	−76.81
	*FZD7*	frizzled class receptor 7	−74.23
	*OR4D9*	olfactory receptor family 4 subfamily D member 9	−73.86
	*PLPPR1*	phospholipid phosphatase related 1	72.62

Note: DPMGs: differentially promoter-methylated genes; CC: clinical cow; SC: subclinical cow; HC: healthy cow; vs.: versus; areaStat: the sum of the *t*-statistics in each CpG, the magnitude of its absolute value represents the degree of methylation difference, the positive value represents hypermethylation, and the negative value represents hypomethylation.

### Gene Ontology Annotation and Kyoto Encyclopedia of Genes and Genomes Pathways Enrichment on the Differentially Methylated Genes and Differentially Promoter-Methylated Genes

**FIGURE 3:**
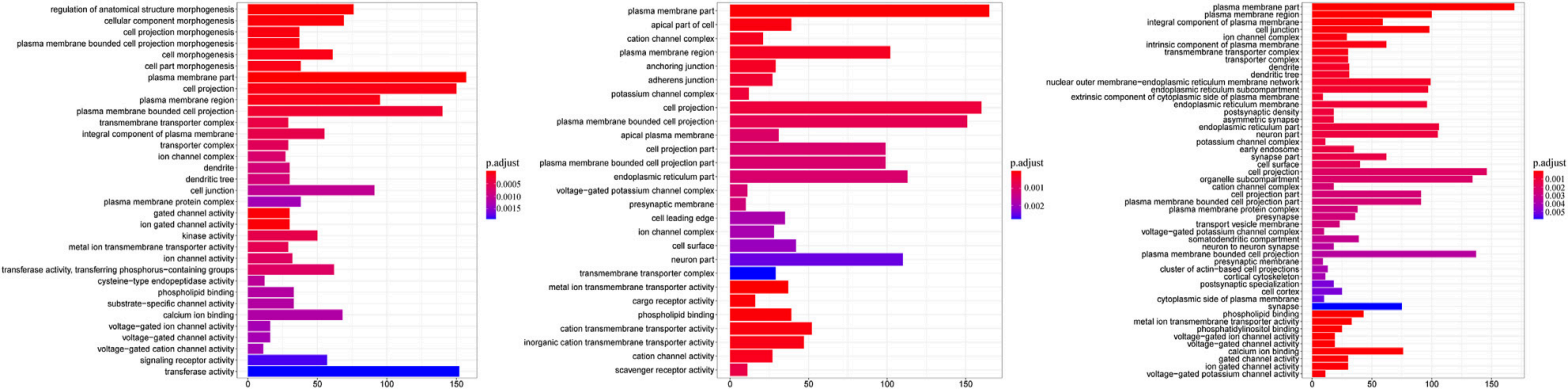
Gene ontology categories enriched for DMGs. The *y*-axis is the name of GO terms; the *x*-axis is the gene number in terms. CC: clinical cow; SC: subclinical cow; and HC: healthy cow.

To further investigate the potential associations of the DMGs and DPMGs with paratuberculosis, we implemented functional enrichment analysis with the clusterprofiler package and found that the DMGs were significantly enriched in 77 GO terms (*q* < 0.05; Figure 3, Supplementary Table S3). From those, the most enriched terms included plasma membrane part (GO:0044459, *q* = 3.51E-06), phospholipid binding (GO:0005543, *q* = 3.80E-05), metal ion transmembrane transporter activity (GO:0046873, *q* = 0.00018), and phosphatidylinositol binding (GO:0035091, *q* = 0.00223).

According to the KEGG analysis, the DMGs were significantly enriched in 181 pathways (*q* < 0.05; Supplementary Table S3) with 95 common pathways across all the comparisons (Supplementary Figure S3). These were mainly related to Th1 and Th2 cell differentiation (bta04658, *q* = 0.03), wnt (bta04310, *q* = 0.00056), TNF (bta04668, *q* = 0.0008), MAPK (bta04010, *q* = 6.98E-08), ECM-receptor interaction (bta04512, *q* = 0.017), cellular senescence (bta04218, *q* = 0.03), calcium (bta04020, *q* = 1.80E-05), and the chemokine signaling pathways (bta04062, *q* = 0.001).

As for DPMGs, no significant GO terms were enriched (*p* > 0.05). However, a total of 92 pathways were enriched (*p* < 0.05, Supplementary Table S3), in which the most significant pathways included oxytocin (bta04921, *p* = 1.46E-05), influenza A (bta05164, *p* = 9.65E-05), prostate cancer (bta05215, *p* = 0.00014), vascular smooth muscle contraction (bta04270, *p* = 0.00015) and the long-term potentiation signaling pathway (bta04720, *p* = 0.00022).

### Identification of Candidate Genes Related to Paratuberculosis With Integrated Whole-Genome Bisulfite Sequencing, Ribonucleic Acid Seq, and Biological Functional Data

Our previous RNA sequencing on the same jejunum samples of the nine Holstein cows used in the present study detected 134 DEGs including 23 between the clinical and subclinical groups, 71 between the subclinical and healthy groups, and 73 between the clinical and healthy groups (*q*<0.05; unpublished data). Of these, 31 functional genes were differentially expressed and differentially methylated among the three comparative groups simultaneously (Supplementary Table S4). Eight genes, calcitonin receptor like receptor (*CALCRL*), tenascin C (*TNC*), GATA binding protein 4 (*GATA4*), CD44 molecule (*CD44*), transglutaminase 3 (*TGM3*), *C-X-C motif chemokine ligand 9* (*CXCL9*), C-X-C motif chemokine ligand 10 (*CXCL10*), and peroxisome proliferator activated receptor gamma (*PPARG*), were significantly enriched in immune-related pathways such as Th1 and Th2 cell differentiation, wnt, TNF, MAPK, ECM-receptor interaction cellular senescence, calcium, and chemokine signaling pathways. Therein, *CXCL10* and *CALCRL* contained two methylated sites in the promoter (Supplementary Figure S4). The Pearson correlation coefficients between the methylated status of the promotor and the mRNA levels (FPKM) of *CXCL10* and *CALCRL* were calculated as -0.07 and -0.56, respectively, indicating downregulation roles of the methylated promoter on gene expression (Supplementary Table S5).

Further, nuclear factor of activated T cells 1 (*NFATC1*), whose methylation of the intron was shown to affect the expression of IL-2 and IL-4 in the effector T cells in previous studies (Akimzhanov et al., 2008; Huang et al., 2011; Christie and Zhu, 2014), was differentially methylated between the MAP-infected and healthy cows and significantly enriched in the Th1 and Th2 cell differentiation pathway (*q* = 0.033).

### Potential Regulatory Mechanism Underlying Paratuberculosis

**FIGURE 4:**
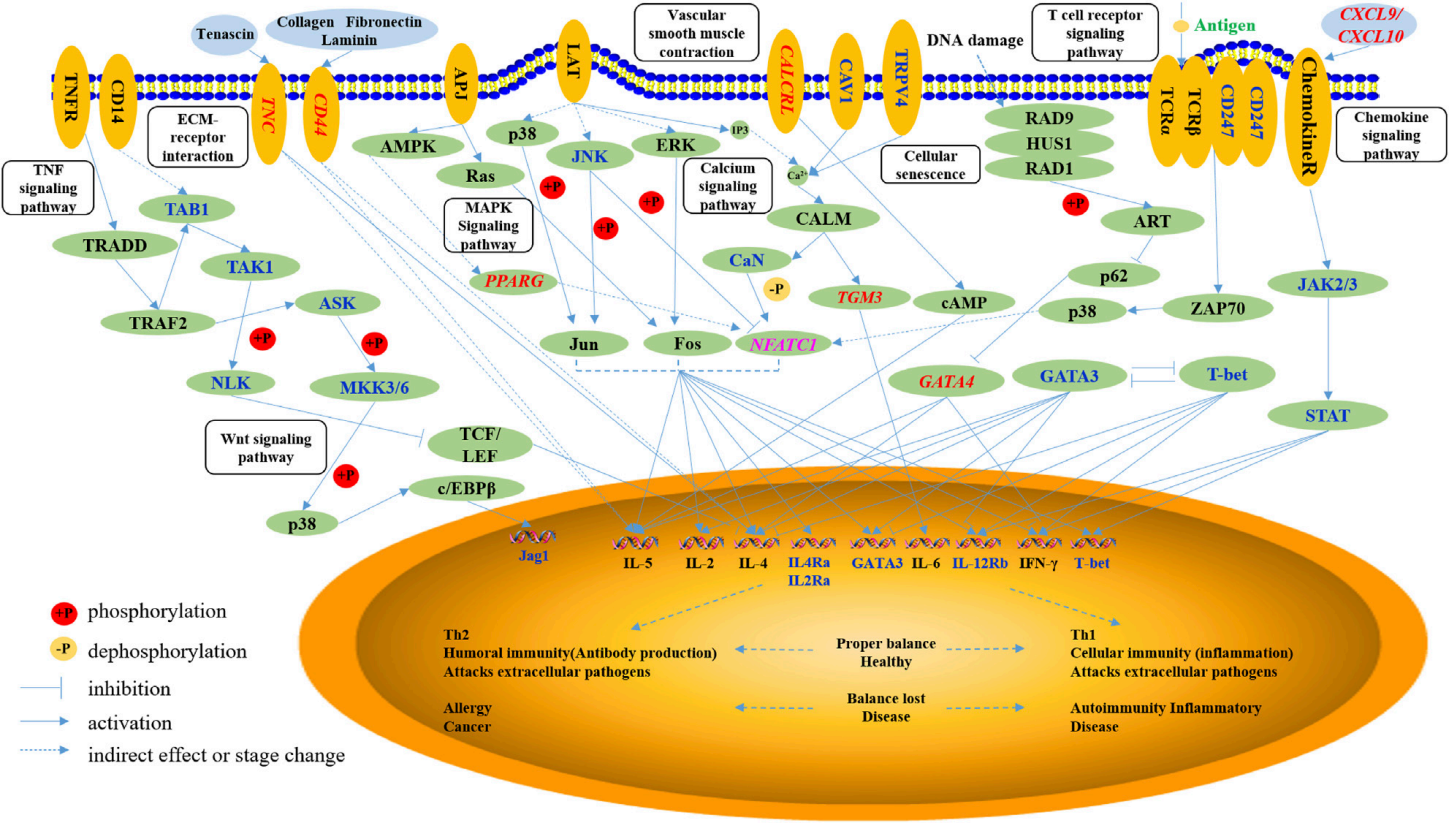
The networks of the immune-related pathways to paratuberculosis. The oval with non-italic characters represents the protein, the oval with italic characters represents the gene, and the small circle represents the small molecule. Orange ellipses are the membrane proteins, the blue ellipses are the extracellular matrix, and green ellipses are the intracellular matrix. Blue and pink characters are the differentially methylated genes, and the red characters are the genes with differential methylation and expression.

Consequently, we inferred the potential of a regulatory mechanism underlying paratuberculosis in dairy cattle. As shown in Figure 4, the differentially methylated *NFATC1*, *TGM3,* and *GATA4* directly affected the expression of immune factors, including IL-5, IL-2, IL-4, IL-4Ra, IL-2Ra, GATA3, IL-6, IL-12Rb, and IFN-*γ* secreted in Th1 and Th2 cells, to maintain an inflammatory response. *NFATC1* elevated the expression of these immune factors through a cell differentiation pathway. *TGM3* activated IL-6 expression via calcium signaling pathways and *GATA4* promoted IL-5, IL-4, and IFN-*γ* through a cellular senescence signaling pathway. *PPARG* regulated *NFATC1* expression through MAPK and calcium signaling pathways thereby modulating immune factors in Th1 and Th2 cells. *CXCL9* and *CXCL10* stimulated chemokine receptor proteins that promoted the JAK2/3 and STAT pathways, ultimately activating immune factors such as IL-12Rb, IFN-*γ*, and T-bet. Membrane proteins encoded by *TNC* and *CD44* modulated the expression of IL-5 and IL-4 through the ECM-receptor interaction signaling pathway and another member protein encoded by *CALCRL* stimulated IL-5 secretion through activation of cAMP in the vascular smooth muscle contraction pathway.

In sum, invasion of MAP into the animal body induced immune responses whereby the above-mentioned nine functional genes stimulated the secretion of immune factors against MAP, indicating the potential regulatory mechanism underlying paratuberculosis in dairy cattle.

## Discussion

In this study, we obtained the integral genome-wide DNA methylation maps of jejunum tissues from clinical, subclinical, and healthy cows with regards to paratuberculosis at the resolution of a single base. We found that the average methylation level was 15–20% in the transcription start site (TSS) and 65–75% in the gene body. These results were similar to the report in humans (TSS: 15–20%, gene body: 60–75%) (Gao et al., 2014), in pigs (TSS: 15–25%, gene body: 65–75%) (Fu et al., 2018; Yang et al., 2021), and even in plants (TSS: 10–20%, gene body: 30–45%) (Song et al., 2013), indicating that a conserved methylation mode existed in trans-species within different species. We observed that most DMRs (46%) were located in the introns region. Del Corvo et al., 2020 also reported that in Italian Simmental, 46% of DMRs in peripheral blood between groups treated by different stress level were located in introns (Del Corvo et al., 2020). This may be because the intron sequence is much longer than the encoding region.

The DNA methylation status of the promoter can affect gene expression *via* changes in chromatin structure or transcription efficiency (Miller and Grant, 2013). In our study, we found that both *CALCRL* and *CXCL10* genes whose promoters contained two methylated sites showed lower expression levels based on our WGBS and RNA-seq data. This may be due to the methylation state slowing down the progress of RNA polymerase during transcription, given that delay of the transcription extension and appearance can inhibit the abnormal transcription initiation (Barry et al., 1993; Hohn et al., 1996; Rountree and Selker, 1997).

Based on integrated information about the DMGs, DEGs, and their biological functions, we identified nine genes as promising candidates for resistance or susceptibility to paratuberculosis in dairy cattle. They were *CALCRL*, *TNC*, *GATA4*, *CD44*, *TGM3*, *CXCL9*, *CXCL10*, *PPARG,* and *NFATC1*. *CALCRL* encodes the membrane receptor CRLR, which is associated with diseases of lymphatic malformation and changes contraction of the vascular smooth muscle resulting in an increase in tissue fluid and thus leads to edema (Dong et al., 2005; Garvey, 2018). *TNC* encodes an extracellular matrix protein, which regulates the expression of immune factors IL-4 and IL-5 and has a critical role in humoral immunity. (Zhou et al., 2019) found that the *TNC* levels of serum correlated strongly with occurrences of colorectal cancer (Zhou et al., 2019). *GATA4* encodes a member of the GATA family of zinc-finger transcription factors that is associated with inflammation induced by DNA damage in mouse embryonic fibroblasts (Reyes et al., 2004; Kang et al., 2015). *CD44* participates in a wide variety of cellular functions including lymphocyte activation and tumor metastasis (Dalerba et al., 2007; Schmitt et al., 2015), and *CD44* plays important roles in ECM-receptor and wnt signaling pathways related to differentiation of the invasion of tumor cells (Hill et al., 2006; Wan et al., 2019) reported that the *CD44* expression influenced susceptibility to colorectal cancer in humans, and that an SNP (rs187115) in *CD44* was associated with an increased risk of colorectal cancer (Wan et al., 2019). *TGM3* encodes an enzyme that is involved in immune regulation of cancer. (Feng et al., 2020) revealed that *TGM3* potentially suppressed cell proliferation through promoting apoptosis and cell cycle regulation and activating phosphorylated AKT serine/threonine kinase to inhibit invasion and metastasis in colorectal cancer cells (Feng et al., 2020). *CXCL9* has a critical role in T cell transport and recruitment of mononuclear cells and granulocytes in the host immune response (Liu et al., 2008; Elia and Guglielmi, 2018). Antonelli et al., 2014; Tokunaga et al., 2018 reported that *CXCL9* expression in epithelial colonic cells interacted with immune factor IFN-γ in ulcerative colitis (Antonelli et al., 2014; Tokunaga et al., 2018). *CXCL10* was associated with chronic inflammation, immune dysfunction, and dissemination of the tumor (Liu et al., 2011). It was reported that the downregulation of *CXCL10* expression was connected with tumor metastasis and recurrence of colorectal cancer, and the increasing expression of *CXCL10* influenced the recruitment of CD8^+^ T lymphocytes to tumor sites (Li et al., 2014; Zheng et al., 2016). *PPARG* decreased the inflammatory response and increased synthesis and release of paraoxonase1, and its activation was shown to inhibit pro-inflammatory genes via trans-repression (Straus and Glass, 2007; Khateeb et al., 2010). *PPARG* was also associated with beta-catenin, an important molecule in colorectal tumor carcinogenesis and C > T mutation in exon 8 that increased the risk of colorectal cancer (Michalik et al., 2004; Tontonoz and Spiegelman, 2008; Lin et al., 2019). *NFATC1* encodes the NFAC1 protein that plays a role in the inducible expression of cytokine genes in T cells. *NFATC1* induced *IL-2* or *IL-4* expression in T cells to regulate the activation differentiation and proliferation of programmed death of T lymphocytes (Huang et al., 2011). In our study, the methylation level of DMRs in *CALCRL*, *TNC*, *TGM3,* and *PPARG* significantly increased with aggravation of the MAP disease. And the *CXCL9*, *CXCL10,* and *NFATC1* methylation levels of HC groups was significantly higher than CC and SC groups, indicating that the decrease of their methylation levels may be related to the occurrence of inflammation. The details of methylation level can be found in Supplementary Table S4.

Further, we have proposed the regulatory mechanism by which the immune response occurs after MAP invades the body, and how these candidate genes regulate immune pathways related to paratuberculosis in dairy cattle. Once MAP invades the bovine body, the immune response will control the infection immediately; nevertheless, it cannot completely kill MAP (Arsenault et al., 2014). In the early stage of infection, the Th1 cell-mediated immune response is rapid, with a small number of bacteria excreted from the animal body at this stage. As the cellular immunity mediated by Th1 cells weakens, the humoral immunity that is mediated by Th2 cells is gradually strengthened; the disease has progressed to the clinical stage (Spellberg and Edwards, 2001). At this stage, both the antibody level and the number of bacteria excreted from the body are significantly increased (Nielsen and Toft, 2006). In our study, we found that the nine candidate genes (*CALCRL*, *TNC*, *GATA4*, *CD44*, *TGM3*, *CXCL9*, *CXCL10*, *PPARG,* and *NFATC1*) participated in the main immune-related pathways associated with paratuberculosis, including Th1 and Th2 differentiation, ECM-receptor interaction, wnt, TNF, MAPK, calcium, vascular smooth muscle contraction, T cell receptor, cellular senescence, and chemokine signaling pathways. These pathways have the ability to influence the secretion of immune factors in T cells, involving IL-5, IL-2, IL-4, IL-4Ra, IL-2Ra, GATA3, IL-6, IL-12Rb, and IFN-*γ*. This implies that they function in transmitting intercellular information, regulating the physiological process of cells and resistance to MAP.

## Conclusion

In this study, we obtained genome-wide methylomes with a single-base resolution of jejunum tissue from Holstein cows in clinical, subclinical, and healthy groups for paratuberculosis, and identified 8,432 differentially methylated genes. With integration of information about DMGs, DEGs, and biological functions, we reported nine promising candidate genes associated with resistance/susceptibility to paratuberculosis: *CALCRL*, *TNC*, *GATA4*, *CD44*, *TGM3*, *CXCL9*, *CXCL10*, *PPARG,* and *NFATC1*. Our findings provide new insights into the regulatory mechanism of bovine paratuberculosis and associated molecular information for gene-based improvements to the health of dairy cattle.

## Data Availability

We have submitted our sequencing data to the NCBI Sequence Read Archive (SRA) and the data has been reserved under BioProject accession PRJNA745069.
