# Supplementary figures and images for: Genome-Wide DNA Methylation Profile in Jejunum Reveals the Potential Genes Associated With Paratuberculosis in Dairy Cattle
Source: Front Genet. 2021 Oct 15;12:735147. doi: 10.3389/fgene.2021.735147 (PMC8554095; doi:10.3389/fgene.2021.735147)

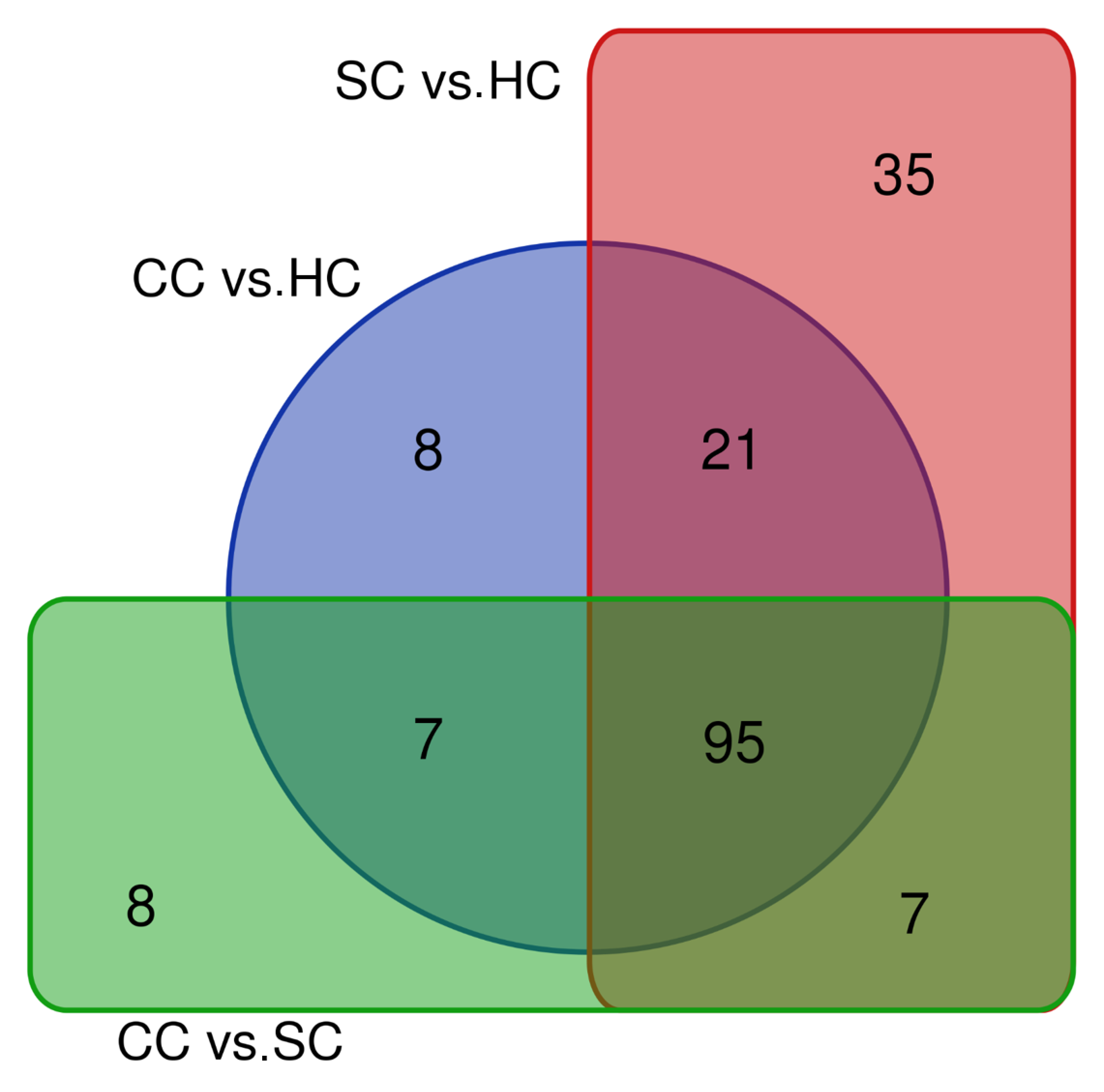

Supplement: Supplementary file 3 [file Image3.TIF]

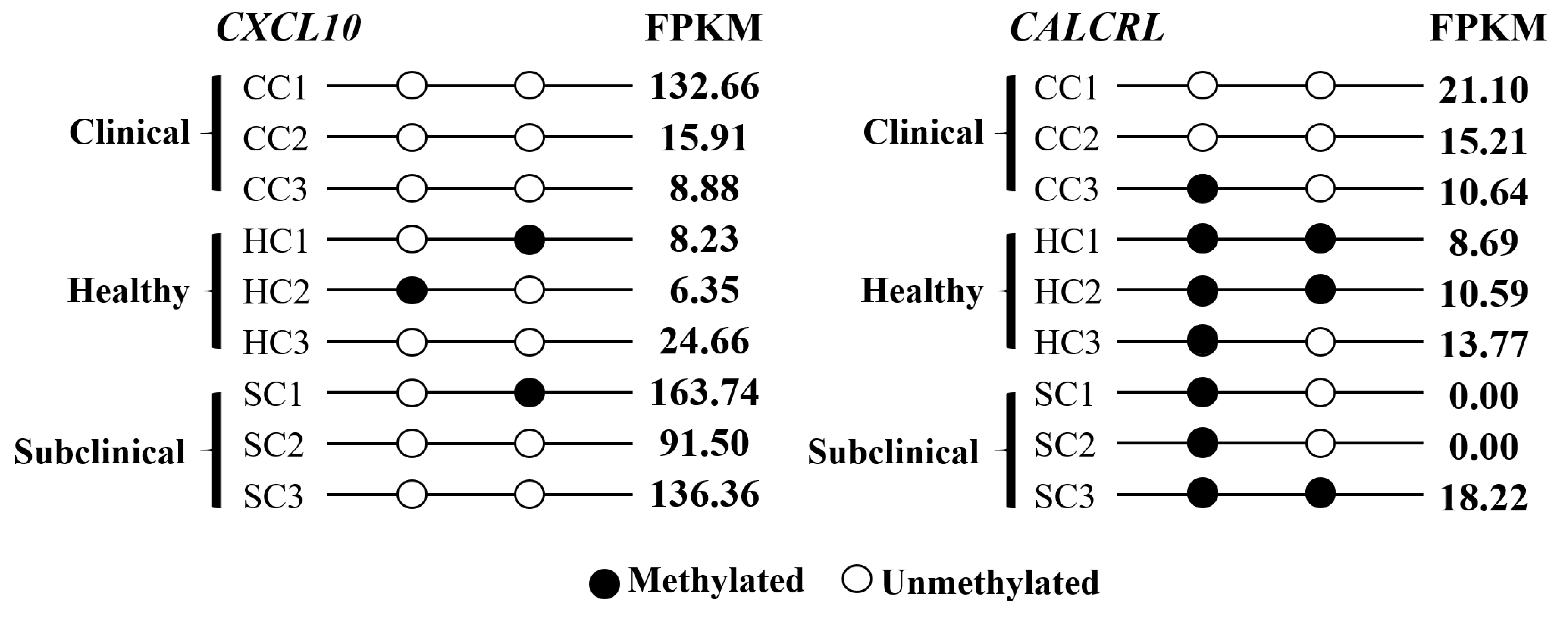

Supplement: Supplementary file 4 [file Image4.TIF]

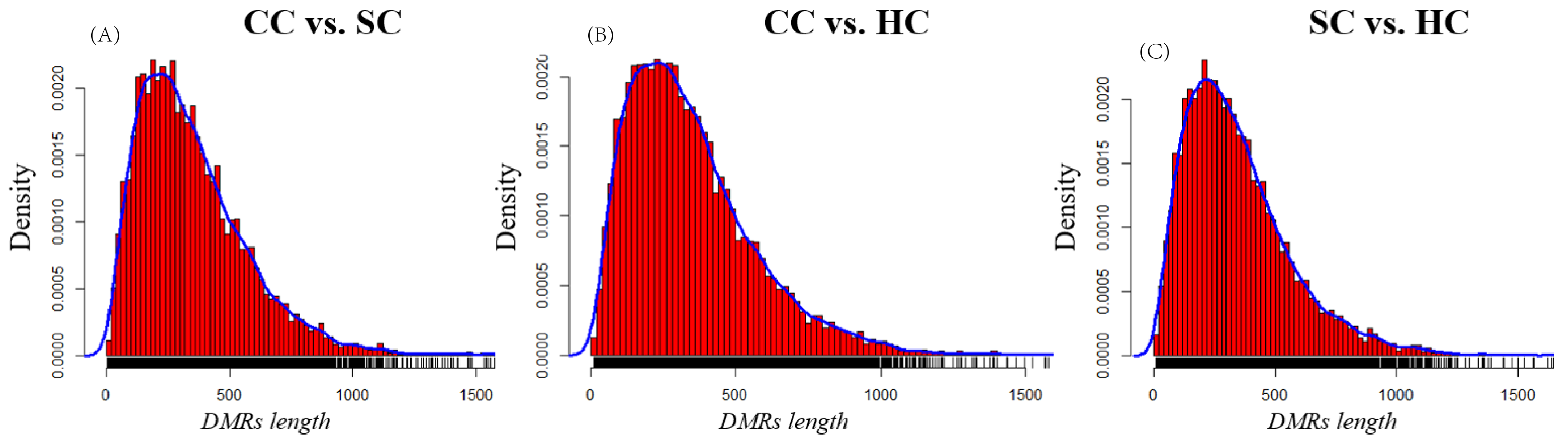

Supplement: Supplementary file 5 [file Image2.TIF]

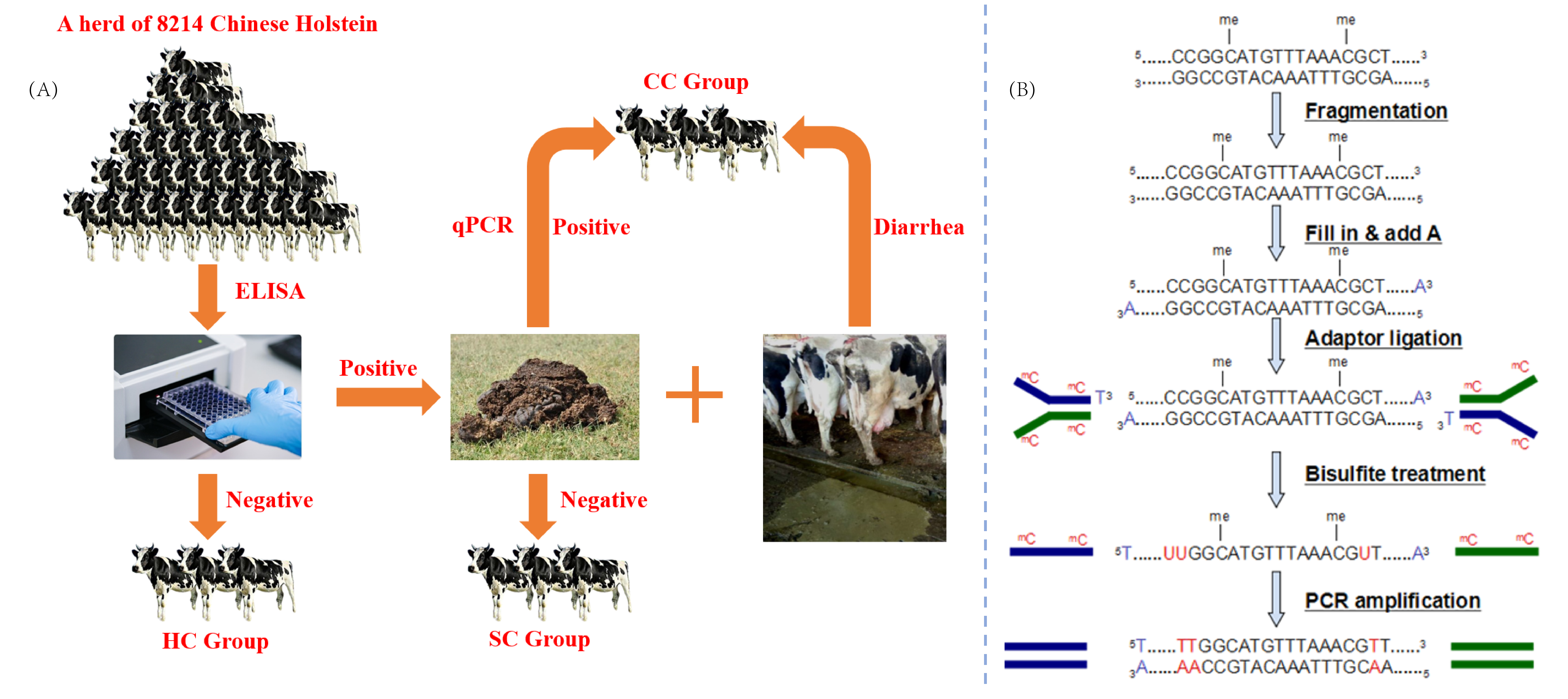

Supplement: Supplementary file 6 [file Image1.TIF]
